# Supplementary material for: Anionic nanoparticle-lipid membrane interactions: the protonation of anionic ligands at the membrane surface reduces membrane disruption
Source: RSC Adv. 2019 May 7;9(25):13992–7. doi: 10.1039/c9ra02462j (PMC9064125; doi:10.1039/c9ra02462j)
Supplement: RA-009-C9RA02462J-s001 [file RA-009-C9RA02462J-s001.pdf]

Electronic Supplementary Material (ESI) for RSC Advances.
This journal is © The Royal Society of Chemistry 2019

Anionic nanoparticle-lipid membrane interactions: the protonation of anionic ligands at the membrane surface reduces membrane disruption

Sebastian Salassi[a], Ester Canepa[b], Riccardo Ferrando[a] and Giulia Rossi[a†]

[a] *Department of Physics, University of Genoa, Via Dodecaneso 33, 16146 Genoa, Italy*
[b] *Department of Chemistry and Industrial Chemistry, University of Genoa, Via Dodecaneso 31, 16146 Genoa, Italy*
[†] rossig@fisica.unige.it

Electronic Supplementary Information

Coarse grained models. The model for the nanoparticle (NP), described and validated in our earlier work[1,2], comprises an atomistic description of the Au core with a coarse-grained (CG) representation of the ligand shell. The CG model is based on the polarizable water MARTINI force field[3,4]. Hydrophobic octane thiol (OT) ligands are described by a chain of two C_1 beads. Anionic 11-mercaptoundecanoic acid (MUA) ligands are described by a chain of 3 hydrophobic C_1 beads and one terminal charged Q_{da} bead. The protonated MUA ligand differ from the charged one by the terminal bead which is a neutral P_3 type. The NP are covered by 30 hydrophobic OT ligands and 30 hydrophilic MUA ligands with a random grafting on the Au core. All the topology files can be found in our online repository[5].

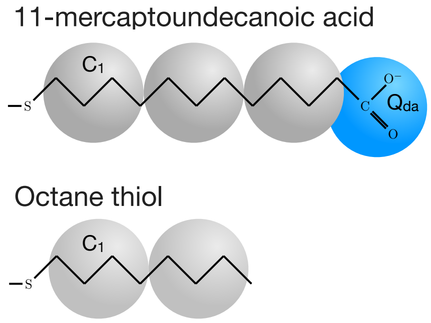

Figure S1. Atomistic structure of the ligands with the corresponding Martini mapping scheme: hydrophobic C_1 beads in gray and negatively charged Q_{da} bead in blue. The protonated MUA ligand differ from the charged one by the terminal beads which is a neutral P_3 type.

Simulation setup. Unbiased and metadynamics[6,7] simulations were run following the simulation setup described in our previous work[1]. In short, one NP was placed in contact with the POPC bilayer (Top left image in Fig. 1). The bilayer is composed by 512 POPC lipids, and the simulation box size is 13x13x18 nm^3. Na^+ counter ions were added to the solution to balance the NP charge.

We performed simulations in the NPT ensemble. We used the velocity–rescale thermostat[8] to set the temperature to 310 K. The pressure was semi-isotopically kept constant to 1 bar with the Berendsen[9] and the Parrinello–Rahman[10] algorithms for the equilibration and production run respectively. We used a timestep of 20 fs. All unbiased simulations were performed with Gromacs 2016.

Metadynamics. Metadynamics simulations were performed following the setup described in our previous work[1]. Briefly, the collective variable is the distance between one terminal group and the membrane center of mass along the membrane normal (which is the *z* axis in our case). The gaussian height and width are

2.48 kJ/mol and 0.06 nm, respectively, and the deposition time is 1 ns. All metadynamics simulations were performed with Gromacs 2016 patched with Plumed 2.3[11].

Constant pH-simulation. In the constant pH-simulations the protonation state of the carboxyl group of the anionic ligands was reassigned based on group pK_a at regular intervals of time. The charge state of each carboxyl terminal bead was reassigned based on its probability $P_{\mathrm{p}}(z)$ to be protonated as a function of its distance from the membrane center of mass (right panel of Fig.2 of the paper). The probability $P_{\mathrm{p}}(z)$ is given by the Boltzmann distribution:

$$P_{\mathrm{p}}(z) = \frac{n_p}{n_{\mathrm{dep}} + n_{\mathrm{p}}} = \frac{n_p}{n_{\mathrm{dep}}} \frac{1}{1 + \frac{n_p}{n_{\mathrm{dep}}}} = \frac{\exp\left(\Delta G^{\mathrm{dep}\rightarrow\mathrm{p}}/k_B T\right)}{1 + \exp(\Delta G^{\mathrm{dep}\rightarrow\mathrm{p}}/k_B T)}$$

Where n_p and n_{dep} are the number of protonated and deprotonated ligands, respectively; and $\Delta G(z)^{\mathrm{dep}\rightarrow\mathrm{p}}$ is the free energy difference between the protonated and deprotonated state. The simulation was splitted in a series of windows, each of length Δt. At the beginning of each window the protonation state of the NP, *i.e.* the total number of deprotonated MUA ligands, is changed by reassigning the protonation state of each MUA ligand: for each terminal bead at distance *z* a random value *p* is extracted from a uniform distribution. If $p < P_p(z)$ then the bead is set protonated; deprotonated otherwise. The number of counter ions in the box is changed accordingly, to maintain neutrality, while the total number of particles is kept constant. If, at the beginning of window *i*, the total number of protonated ligands increases with respect to window *i-1*, the excess of counter ions (Q_{da} Martini beads, positively charged) are transformed into neutral Q_{da} beads; *vice-versa* if the number of deprotonated ligands increases.
We simulated a total of about 23 µs in 3 independent simulation, using in house scripts to reassign the protonation state at the beginning of each window. These MD simulations were performed with Gromacs 2018.

Extra constant-pH simulations. We have performed two more constant-pH simulations, which are not described in the main paper. One run was initialized from a configuration in which the NP has about 10 ligands anchored to the opposite leaflet, as shown in the second figure of the top panel of Fig. 4, and Δt is set to 30 ns. The third run was initialized with a configuration in which the NP has 14 anchored ligands, as shown in the third figure of top panel of Fig. 4, and Δt = 20 ns. The plots of the protonated and anchored ligands and the distance of the NP from the membrane COM is shown, respectively, in Fig. S3 and S4. We observe the same behavior as in the first run: in some cases, the NP reaches the fully immersed configuration with half ligands anchored to both leaflet and the distance from the membrane COM happens to become negative. Also, increasing the duration of each windows (Δt) did not result in any qualitative difference on the NP behavior.

Contact Analysis. The number of water contacts with the biased charged terminal group were obtained with the gromacs *mindist* tool with a cut-off distance of 0.6 nm.

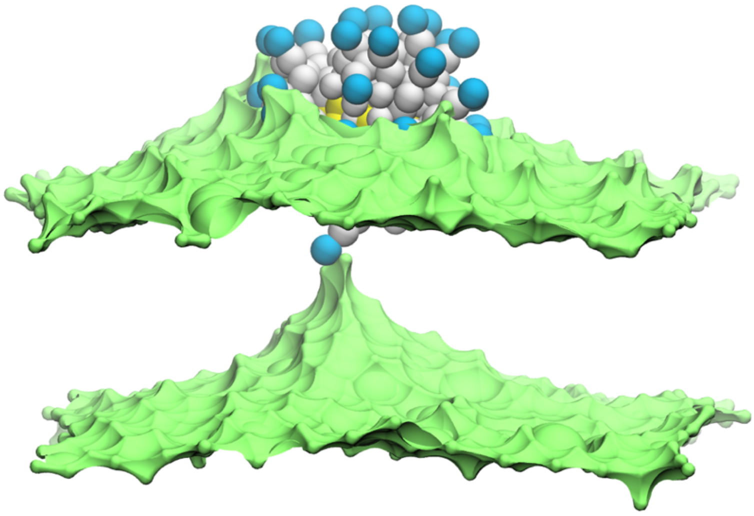

Figure S2. Membrane deformation during the anchoring transition of the anionic ligand. Blue beads represent the anionic terminal beads. Hydrophobic ligand beads in white, lipid heads in green (surface representation). Hydrophobic core of the membrane and water beads are not shown for clarity.

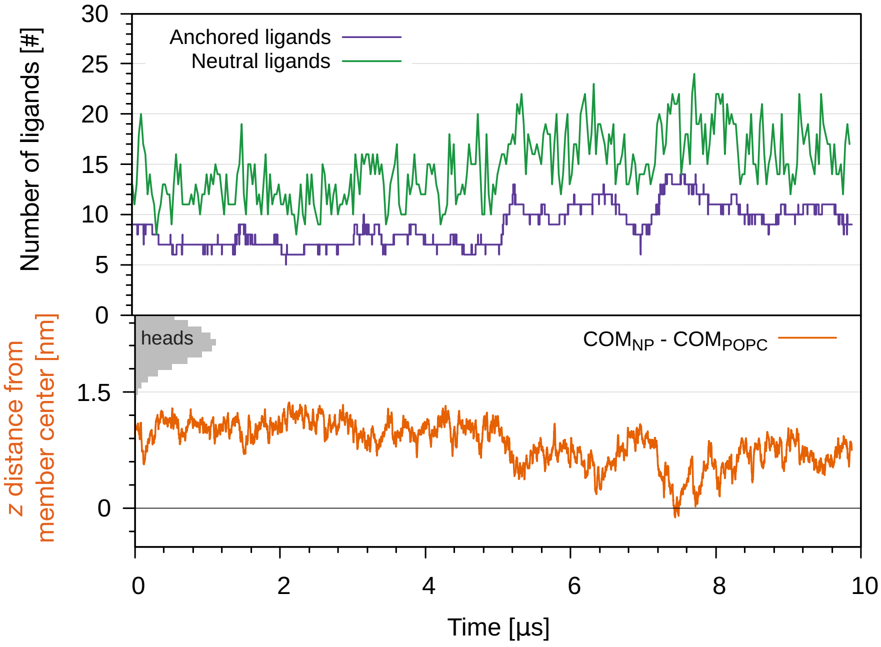

Figure S3. Constant-pH simulation initialized from a configuration in which the NP has about 10 ligands anchored to the opposite leaflet. Top panel: the number of anchored ligands (violet) and protonated ligands (green) as a function of the simulation time. Bottom panel: *z* distance between the membrane center and the NP center of mass as a function of the simulation time. The shaded grey areas indicate the distribution of lipid heads of the entrance leaflet.

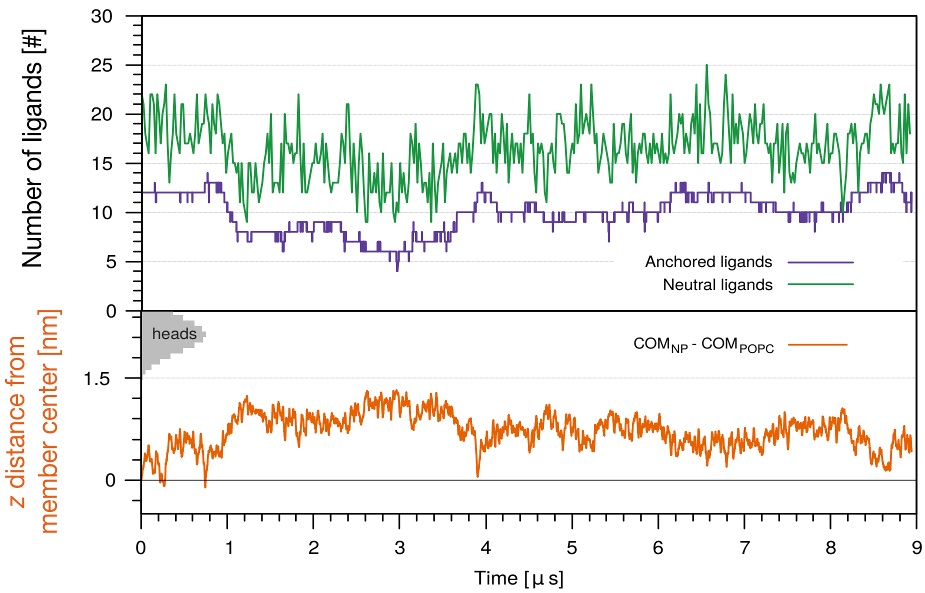

Figure S4. Constant-pH simulation initialized from a configuration in which the NP has about 15 ligands anchored to the opposite leaflet. Top panel: the number of anchored ligands (violet) and protonated ligands (green) as a function of the simulation time. Bottom panel: *z* distance between the membrane center and the NP center of mass as a function of the simulation time. The shaded grey areas indicate the distribution of lipid heads of the entrance leaflet.

References

1 S. Salassi, F. Simonelli, D. Bochicchio, R. Ferrando and G. Rossi, *J. Phys. Chem. C*, 2017, **121**, 10927–10935.

2 F. Simonelli, D. Bochicchio, R. Ferrando and G. Rossi, *J. Phys. Chem. Lett.*, 2015, **6**, 3175–3179.

3 S. J. Marrink, H. J. Risselada, S. Yefimov, D. P. Tieleman and A. H. de Vries, *J. Phys. Chem. B*, 2007, **111**, 7812–7824.

4 S. O. Yesylevskyy, L. V. Schäfer, D. Sengupta and S. J. Marrink, *PLoS Comput. Biol.*, 2010, **6**, e1000810.

5 BioMembNP repository, https://bitbucket.org/biomembnp/biomembnp.

6 A. Laio and M. Parrinello, *Proc. Natl. Acad. Sci. USA*, 2002, **99**, 12562–12566.

7 A. Laio and F. L. Gervasio, *Reports Prog. Phys.*, 2008, **71**, 126601.

8 G. Bussi, D. Donadio and M. Parrinello, *J. Chem. Phys.*, 2007, **126**, 014101.

9 H. J. C. Berendsen, J. P. M. Postma, W. F. Van Gunsteren, A. DiNola and J. R. Haak, *J. Chem. Phys.*, 1984, **81**, 3684.

10 M. Parrinello and A. Rahman, *J. Appl. Phys.*, 1981, **52**, 7182–7190.

11 G. A. Tribello, M. Bonomi, D. Branduardi, C. Camilloni and G. Bussi, *Comput. Phys. Commun.*, 2014, **185**, 604–613.
